# Genome-Based Identification of Heterotic Patterns in Rice

**DOI:** 10.1186/s12284-017-0163-4

**Published:** 2017-05-19

**Authors:** Ulrike Beukert, Zuo Li, Guozheng Liu, Yusheng Zhao, Nadhigade Ramachandra, Vilson Mirdita, Fabiano Pita, Klaus Pillen, Jochen Christoph Reif

**Affiliations:** 1 Department of Breeding Research, Leibniz Institute of Plant Genetics and Crop Plant Research (IPK) Gatersleben, Corrensstraße 3, 06466 Stadt Seeland, Germany; 2 Bayer Bioscience, 500081 Hyderabad, India; 3 European Wheat Breeding Center, Bayer Crop Science, Am Schwabenplan 8, 06466 Stadt Seeland, Germany; 4 Biometrics and Breeding Research US, Bayer Crop Science, 407 Davis Dr., Morrisville, NC USA; 5 K. Pillen, Chair of Plant Breeding, Martin-Luther-University Halle-Wittenberg, Betty-Heimann-Str. 3, 06120 Halle/Saale, Germany

**Keywords:** Heterotic Groups, Heterotic Pattern, Hybrid Rice, Genome-Wide Predictions

## Abstract

**Background:**

Hybrid rice breeding facilitates to increase grain yield and yield stability. Long-term success of hybrid breeding depends on the recognition of high-yielding complementary heterotic patterns, which is lacking in crops like rice.

**Result:**

The main goal of this study was to evaluate the potential and limits to use genomics for establishing heterotic patterns in rice. For this purpose, data of a commercial hybrid rice breeding program targeted to India was analyzed, including 1,960 phenotyped hybrids from three market segments and 262 genotyped parental lines. Our cross-validation study revealed that grain yield of all potential single-crosses can be accurately predicted. Based on the full matrix of hybrid performances, high-yielding heterotic patterns were identified. These heterotic patterns increased grain yield up to 9% compared to the currently employed groups. Heterotic groups of around 14 individuals reflect a good compromise between long-term and short-term selection response.

**Conclusions:**

Our findings clearly underlined the benefits of a genome-based establishment of heterotic patterns in rice as a requirement for a sustainable long-term success of hybrid rice breeding.

**Electronic supplementary material:**

The online version of this article (doi:10.1186/s12284-017-0163-4) contains supplementary material, which is available to authorized users.

## Background

Rice belongs to the three leading food crops providing the majority of calories to feed the world (International Rice Research Institute 2013). The demand for rice is raising steadily due to changes in customer priorities and population growth (Khush 2013). Thus, rice production has to be increased. Unfortunately, the potential of expanding the cultivated area is limited (Khush 2005) and, therefore, rice production has to be increased by enhancing yields per area (Khush 2013). Nevertheless, worldwide selection gain in rice is only 1.0% per year and much lower than the required increase in yield potential of 2.4% (Ray et al. 2013). Thus, there exits the strong need to develop and implement improved breeding tools (Phillips 2010; Zhu et al. 2016). A promising approach to boost yield per area consists in hybrid breeding, which has been successfully applied in rice improvement for some target regions such as China (Khush 2005; Khush 2013).

The advantages of hybrid versus line breeding are the exploitation of heterosis resulting in higher grain yield (Xu et al. 2014) and an enhanced yield stability (Longin et al. 2012). Furthermore, hybrid breeding simplifies to stack major dominant genes and results in substantial return on investments, which is required to refinance future breeding progress (Longin et al. 2012). Hybrid rice breeding programs were initiated in China in 1964 based on a cytoplasmic male sterility (CMS) system. The first hybrid rice variety was released in 1976 (Barclay 2010). In addition to CMS, photoperiod sensitive male sterility has been exploited to produce hybrid seeds on a large scale (Chen and Liu 2014; Li et al. 2007). Rice grain yield per area increased through hybrid breeding by approximately 40% during the past three decades (Zhu et al. 2016). As a consequence, hybrid varieties covered more than 50% of China’s total rice growing area in 2003 (Cheng et al. 2007a). Moreover, the success of hybrid rice in China encouraged embarking on a national program for the development of hybrid rice for India in 1989 (Viraktamath 2010).

The optimum exploitation of heterosis requires that the available germplasm is structured into genetically diverse heterotic groups. A heterotic group refers to a collection of genotypes resulting in similar hybrid performance when crossed with individuals belonging to a complementary and genetically distinct germplasm group. A specific combination of two heterotic groups leading to high-yielding hybrids is defined as heterotic pattern (Melchinger and Gumber 1998). Hybrid breeding based on the concept of heterotic patterns leads to more pronounced variance of the breeding values in contrast to the variance of the dominance deviations, which enhances recurrent selection gain (Reif et al. 2007).

Heterotic patterns have been established in the past either considering hybrid seed production traits such as seed yield of the female lines and high pollination capability of the male lines (Reif et al. 2005) or they have been developed empirically by testing combining ability among available germplasm (Fischer et al. 2010). The latter approach is afflicted by the large number of possible hybrid combinations among available elite inbred lines. To solve this challenge, Zhao et al. (2015) developed a three-step approach to search for heterotic patterns: (1) The performance of all possible single-cross combinations is determined through genome-wide or metabolite-based hybrid predictions. (2) The predicted hybrid performances are used to identify superior heterotic patterns based on a simulated annealing algorithm. (3) The optimum size of the heterotic pattern is determined balancing the expected short- and long-term selection.

The genetically distant subgroups of *indica* and *japonica* have been suggested as a promising heterotic pattern for hybrid rice breeding (Cheng et al. 2007a). Nevertheless, fertility barriers hamper so far the exploitation of this heterotic pattern (Ikekashi and Araki 1984; Liu et al. 1996). Several approaches have been used to reduce the sterility occurring when crossing *indica* and *japonica* (Guo et al. 2016; Ouyang et al. 2010), but none of them has been successfully implemented into hybrid rice breeding programs. The search for heterotic groups within the major germplasm pools is limited to studies based on tropical *indica* rice (Wang et al. 2014; Xie et al. 2013). Moreover, previous studies used molecular marker-based genetic distances as proxy of the heterosis or hybrid performance, which is unreliable for unrelated parental inbred lines (Melchinger 1999; Xu et al. 2014; Xu et al. 2016). Thus, other approaches exploiting for example genome-wide hybrid predictions are needed to recognize promising heterotic groups for hybrid rice breeding.

Our study is based on genomic and phenotypic data of 1,960 rice hybrids adapted to the Indian sub-continent, which have been evaluated for grain yield in two to four locations. The hybrids were grouped based on grain size, shape and appearance into three market segments: Long grain (LS) segment with grain type of long slender, length of larger than 6 mm, length/breadth ratio of larger than 3; Medium (MM) grain segment with grain type of medium slender, length of less than 6 mm, length/breadth ratio of 2.5 to 3.0 mm; Short (SS) grain segment with grain type of short slender, length of less than 6 mm, length/breadth ratio of larger than 3. Our main goal was to evaluate the potential and limits of genome-based establishment of heterotic patterns in rice. In particular, the objectives were to (1) investigate the accuracy for genome-wide prediction of hybrid performance in rice, (2) evaluate the benefits of heterotic patterns identified with a simulated annealing algorithm, and (3) assess the optimal size of the heterotic patterns balancing short- and long-term selection gain.

## Results

### Variance due to specific combining ability effects played a prominent role in hybrid rice populations

**Fig. 1:**
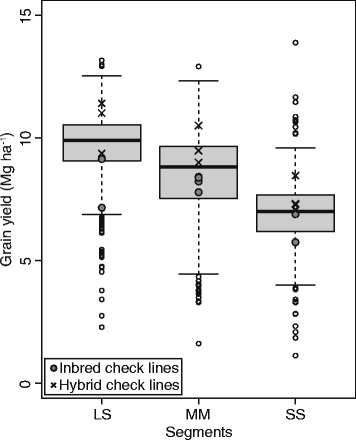
Best linear unbiased estimations for grain yield performance of hybrids and checks of the market segments LS, MM, and SS

We observed a wide range of Best Linear Unbiased Estimations leading to significant (*P* < 0.001) genetic variances within all three market segments (Fig. 1). In total, 19, 4, and 10% of the hybrids significantly (*P* < 0.05) outperformed leading commercial varieties for the market segments LS, MM, and SS, respectively. Genetic variance components involving general combining ability effects ${\sigma}_{GCA}^2$ showed higher values for male than for female lines (Table 1). The variance of specific combining ability effects ${\sigma}_{SCA}^2$ was for all segments significantly (*P* < 0.05) larger than zero and contributed on average to 42% of the total genetic variance. Heritability estimates ranged from 0.26 to 0.61.

**Table 1:** Second degree statistics for hybrid grain yield (Mg ha^−1^) experiments in market segments LS, MM, and SS performed in three, two, and four environments, respectively

Source	LS	MM	SS
${\upsigma}_{\mathrm{Genotype}}^2$	0.99***	0.53***	0.81***
${\upsigma}_{\mathrm{GCA}\ \mathrm{male}}^2$	0.30**	0.27	0.24***
${\upsigma}_{\mathrm{GCA}\ \mathrm{female}}^2$	0.24**	0.13	0.11***
${\upsigma}_{\mathrm{SCA}}^2$	0.45***	0.13*	0.46***
${\upsigma}_{\mathrm{Genotype}\ \mathrm{x}\ \mathrm{Location}}^2$	1.48***	2.10***	0.90***
${\upsigma}_{\mathrm{GCA}\ \mathrm{male}\ \mathrm{x}\ \mathrm{Location}}^2$	0.88***	1.39***	0.33***
${\upsigma}_{\mathrm{GCA}\ \mathrm{female}\ \mathrm{x}\ \mathrm{Location}}^2$	0.11***	0.26***	0.12***
${\upsigma}_{\mathrm{SCA}\ \mathrm{x}\ \mathrm{Location}}^2$	0.49***	0.45**	0.45***
${\upsigma}_{\mathrm{error}}^2$	0.59	0.97	1.34
h^2^	0.59	0.26	0.61

Probability level: *** < 0.001, ** < 0.01, * < 0.05, not assigned not significant

### Analyses of population structure revealed genetically distinct parental pools for market segments MM and SS but not for LS

**Fig. 2:**
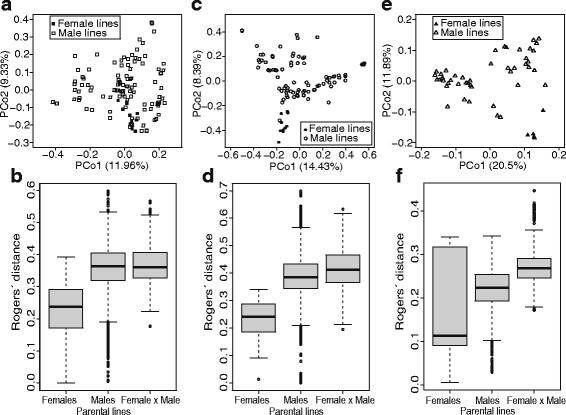
Principal coordinate analyses of parental lines (**a**) for market segments LS, (**c**) MM, and (**e**) SS with relating distribution of Rogers’ distances within and between parental pools (**b**) for market segments LS, (**d**) MM, and (**f**) SS

The analyses of linkage disequilibrium (LD) revealed a fast decay of LD with increasing physical distance (Additional file 1: Figure S1). The sharp decay of LD underlines the potential of using the populations for high-resolution genome-wide association mapping. The population structure was examined by applying principal coordinate analyses. The analyses of the 262 genotyped lines revealed absence of a population structure among market segments (Additional file 1: Figure S2). Despite this, differences in quality requirements are large and parents are used only rarely across segments MM, SS, and LS. Thus, we analyzed in the following each market segment separately. In segment LS, male and female lines did not clustered separately (Fig. 2). This was further substantiated by the distributions of Rogers’ distances with highest values between male lines as well as between male and female lines (Fig. 2b). In contrast, for segments MM and SS, principal coordinate analyses and distributions of pairwise Rogers’ distances revealed genetically distinct parental pools (Fig. 2c, d, e and f).

### Hybrid performance can be predicted with high accuracy

**Fig. 3:**
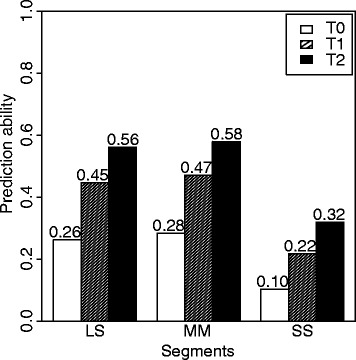
Prediction ability of grain yield performance for different subgroups of market segments LS, MM, and SS

The predicted hybrid performance values of the full diallel matrix formed the basis to search for promising heterotic patterns. We used a chess-board like cross-validation strategy to evaluate the prediction ability of the hybrid performance (Additional file 1: Figure S3). The hybrids were split into an estimation set and the test groups T2, T1, and T0 with decreasing relationship to the estimation set. The accuracy observed in the T2 scenario is relevant for compiling the full hybrid performance matrix, because the parents of all predicted hybrids were evaluated in other single-cross combinations. The prediction abilities ranged for the T2 scenario from 0.33 to 0.58 (Fig. 3). The prediction accuracies were estimated by standardizing the prediction abilities with the square root of heritabilities and amounted to 0.72 averaged across the three market segments.

### Detected heterotic patterns are stable across varying group sizes

**Fig. 4:**
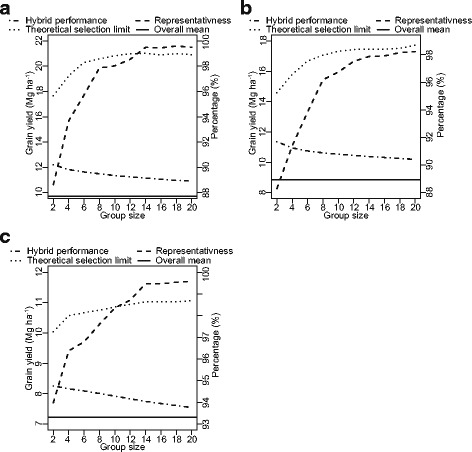
Short- (Hybrid performance) and long-term success (Representativeness, Theoretical selection limit) in dependence of heterotic group size for market segments (**a**) LS, (**b**) MM, and (**c**) SS

We used the predicted performances of all potential single-cross hybrids in order to identify high-yielding heterotic patterns with population sizes ranging from two to 20 lines using a previously developed simulated annealing algorithm. In this search, we assumed that lines can be clustered irrespective to their restoration ability. For all three segments, selection and clustering of lines into heterotic groups were stable across the full range of examined population sizes (Additional file 1: Table S1). Interestingly, only few female lines were selected to establish heterotic patterns: In market segment MM, no female line was selected. In the market segments LS and SS, 38 and 40% of the females were selected, respectively. The selected heterotic patterns substantially outperformed the overall mean with a maximum difference ranging from 14% for market segment SS to 28% for market segment MM (Fig. 4a, b and c).

**Fig. 5:**
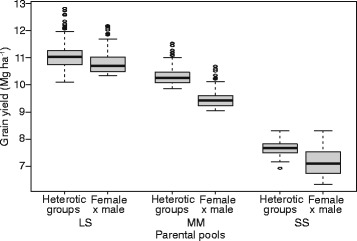
Distribution of predicted hybrid performances for 256 best performing female x male crosses in comparison to heterotic groups of size 16

The performances of the identified heterotic patterns were compared with the average hybrid performance of crosses between male and female lines. In order to make a fair comparison, we focused on a standardized population size of 256 hybrids. The hybrids of the identified heterotic patterns surpassed the superior female and male single crosses by 2, 9, and 7% for market segments LS, MM, and SS, respectively (Fig. 5).

### Short- and long-term success of the identified heterotic patterns

We used the theoretical selection limit as a parameter to assess the long-term success of the detected heterotic groups. The selection limit increased with growing heterotic group size and plateaued at around 14 individuals across the different market segments (Fig. 4a, b and c). We further assessed the long-term success of the heterotic patterns by estimating genetic representativeness, which also plateaued at around 14 genotypes. In contrast, the short-term success assessed as the population mean was highest at small population sizes and decreased nearly linearly with increasing size of the heterotic patterns.

## Discussion

More than 50% of the total cultivated rice area in China is grown with hybrid varieties, which was enabled by substantial investments in rice research and breeding (Cheng et al. 2007b). Encouraged by the success of hybrid rice in enhancing the rice production and productivity in China, India initiated a national program for the development and large scale adaption of hybrid rice in 1989 (Viraktamath 2010). Plant breeders in the government and private sectors have launched hybrid varieties for different states in India. These hybrids have a 10 to 44% higher yield compared to popular high-yielding line varieties (Wanjari et al. 2006). Thus, a good beginning has been made by ushering in to an era of hybrid rice breeding and production in India (Spielman et al. 2013). Long-term success of hybrid breeding depends on the establishment of complementary heterotic groups (Reif et al. 2007; Zhao et al. 2015). Nevertheless, heterotic groups in rice are not clearly defined (Xie et al. 2012). This encouraged us to assess the potential and limits of a recently developed three-step approach to search for high-yielding heterotic patterns using data of a commercial hybrid rice breeding program. The hybrid rice breeding program is centered on a CMS hybrid seed production strategy. Lines have been clustered based on their restoration ability into female and male groups. The molecular analyses revealed for two of the examined rice market segments that male and female pools were genetically divergent (Fig. 2). Two major restorer genes *Rf3* and *Rf4* are known for the underlying CMS-WA cytoplasm (Zhang et al. 1997; Zhang et al. 2002). Thus, a conversion of female into male lines or vice versa can be realized but is sometimes hampered by modifier genes influencing the penetrance of the restorer genes. Despite this, we assumed in our study that the hybridization system is not restricting the grouping of lines.

### Hybrids significantly surpassed yield performance of commercial line varieties

The yield advantage of rice hybrids over released line varieties, which is often denoted as commercial heterosis, was reported to be in the range of 10 to 20% (Cheng et al. 2007a; Huang et al. 2016; Wang et al. 2014; Xie et al. 2013). We estimated the magnitude of the commercial heterosis by contrasting grain yield of the phenotyped hybrids with the performance of the highest-yielding line variety included in the trials. The commercial heterosis amounted to 25, 34, and 37% for the segments LS, MM, and SS, respectively (Fig. 1). The slightly higher values compared to the previously reported ones (Cheng et al. 2007a; Huang et al. 2016; Wang et al. 2014; Xie et al. 2013) can be explained by the moderate phenotyping intensity and the augmented design used in our study with unreplicated evaluation of hybrids but replicated evaluation of line varieties. The different allocation of resources for checks and hybrids was caused by restrictions in hybrid seed production. Summarizing, our findings underlined the yield advantage of hybrids over inbred lines and underpinned the potential to boost grain yield through hybrid rice breeding.

### Pronounced variance of specific combining ability effects

A predominance of ${\sigma}_{GCA}^2$ over ${\sigma}_{SCA}^2$ leads to an accurate prediction of the hybrid performance based on GCA effects (Zhao et al. 2013). The experimental resources, which need to be installed to estimate GCA effects, are a linear function of the number of parents. This is in contrast to the resources required to estimate SCA effects, which are a quadratic function of the number of parents.

The incomplete factorial mating designs for the market segments LS, SS, and MM differed in the number of non-phenotyped hybrids (Additional file 1: Figure S4). Nevertheless, this should not bias the estimates of the variance components as we can assume missing at random (Little and Rubin 2002). A favorable ratio of ${\sigma}_{GCA}^2$ versus ${\sigma}_{SCA}^2$ is expected in crops with genetically divergent heterotic groups such as in hybrid maize breeding (Reif et al. 2007). In contrary to this, we observed that ${\sigma}_{SCA}^2$ amounts to 25 to 57% of the total genetic variance in the hybrid populations for the three market segments (Table 1). The ${\sigma}_{SCA}^2$ is higher compared to previous estimates in other selfing species such as wheat (Zhao et al. 2015) or barley (Philipp et al. 2016) and can be explained only partially by a lack of genetic divergence between the parental lines (Fig. 2). Another reason for the low estimates of ${\sigma}_{GCA}^2$ is the reduced diversity in the pool of female lines, which is most likely due to the bottleneck in selecting suitable maintainer lines. The relevance of ${\sigma}_{SCA}^2$ indicates that it is challenging to identify superior hybrid combinations based on GCA effects. Moreover, only slow recurrent selection gain is expected when interpreting ${\sigma}_{GCA}^2$ as an estimate of the variance of the breeding values.

### High accuracy observed for genome-wide hybrid prediction

We implemented ridge regression best linear unbiased prediction (RR-BLUP) of the hybrid performances considering additive and dominance effects (Zhao et al. 2014). The ability of prediction was evaluated applying a chess-board like cross-validation scenario (Additional file 1: Figure S3). The prediction ability averaged across the three market segments increased with increasing relatedness between the estimation and test sets from 0.21 for the T0 scenario to 0.49 for the T2 scenario (Fig. 3). These findings confirmed the high relevance of relatedness as the driving force of the prediction ability (Desta and Ortiz 2014; Zhao et al. 2015).

The prediction accuracy was estimated by standardizing the prediction ability with the square root of heritability estimates to compare our findings across market segments and also with other studies. Prediction accuracies were comparable for segment LS and MM but lower for segment SS (Fig. 3). This difference can be explained by a smaller population size (Additional file 1: Table S2) in combination with a more unbalanced factorial mating design (Additional file 1: Figure S4) for segment SS than LS and MM. Zhao et al. (2015) have shown using a resampling strategy based on 1,604 wheat hybrids that both the number of parents used in the factorial mating design and the balanced representation of parental lines used to generate hybrids are important factors driving the prediction accuracy.

Previous studies observed that the prediction accuracies were smaller for self-pollinating than for outcrossing crops (Philipp et al. 2016; Massman et al. 2012; Technow et al. 2014). Moreover, prediction accuracies were higher for crops with clearly defined heterotic groups versus crops with absence of heterotic groups (Philipp et al. 2016). In line with these expectations, we observed prediction accuracies, which were slightly smaller than reported previously for maize hybrids (Massman et al. 2012) and similar to those reported for wheat (Zhao et al. 2015) and rice hybrids (Xu et al. 2014; Xu et al. 2016). Summarizing, genome-wide prediction is a useful and appropriate method to predict hybrid performances in rice.

### The identified heterotic patterns outyielded the currently used hybrid populations

The search for promising heterotic patterns, which maximizes the grain yield in the hybrid populations, was performed using a simulated annealing algorithm based on the predicted hybrid performance matrices. The detected heterotic patterns outyielded the population means by 14% for SS to 28% for LS and MM (Fig. 4). The lower advantage of SS compared with LS and MM can be explained by the limited genetic diversity of the SS segment (Fig. 2). Moreover, the detected heterotic patterns resulted in a yield improvement of 7 to 35% compared to hybrids between the existing male and female pools (Fig. 5). Interestingly, male lines were selected more frequently for the heterotic patterns than female lines. This finding is in line with those reported by Huang et al. (2016), who observed also a higher yield potential of male parents either pointing to more beneficial alleles carried by male than female lines or to a bottleneck in selecting stable maintainer lines.

### Compromise between short- and long-term success was accomplished with heterotic groups each including 14 lines

The efficiency and success of heterotic patterns can be assessed by short- and long-term response to selection (Zhao et al. 2015). Short-term success is maximized by a high selection intensity choosing only the very best performing parental lines. In contrast, long-term success increases with increasing diversity and heterotic group size. Heterotic group sizes should reflect a compromise between short- and long-term successes.

Short-term selection response was assessed based on the hybrid performance of the selected heterotic pattern. We ignored a combination of hybrid performance and expected selection gain, i.e., the usefulness criterion, which was previously used (Zhao et al. 2015) because the usefulness criterion is mainly influenced by the hybrid performance. The assessment of long-term selection gain was based on the parameters of theoretical selection limit and genetic representativeness as suggested previously (Zhao et al. 2015). The genetic representativeness is an index estimating the proportion of specific genomes within the full population represented by the identified heterotic groups (Druet et al. 2014). The theoretical selection limit denotes the maximum hybrid performance realized by reciprocal recurrent selection based on the detected heterotic groups.

We observed that the parameters of long-term response plateaued at approximately 14 genotypes per heterotic group (Fig. 4). This group size was also related to an acceptable level of short-term selection gain; thus, representing a good compromise between short- and long-term selection response. Zhao et al. (2015) reported a similar heterotic group size of 16 individuals per group achieving a suitable balance between short-term and long-term selection gain for European hybrid wheat breeding. Summarizing, a beneficial hybrid breeding in rice can be realized with a heterotic group size of 14 parents, which is connected to a suitable level of short-term as well as long-term selection gain.

## Conclusions

Hybrid breeding and the effective utilization of heterosis crucially depends on the identification of heterotic patterns. In this study, we have evaluated a three-step approach to detect heterotic patterns in rice using data of a commercial hybrid breeding program. Our findings revealed that hybrid rice breeding based on the identified heterotic patterns holds the potential to boost grain yield and represents an important step for the long-term success of hybrid rice breeding.

## Methods

### Plant material

The plant material included rice hybrids from the breeding program of Bayer Crop Science, adapted to the Indian sub-continent. The hybrids were grouped based on grain size, shape and appearance into three market segments: Long grain (LS) segment with grain type of long slender, length of larger than 6 mm, length/breadth ratio of larger than 3; Medium (MM) grain segment with grain type of medium slender, length of less than 6 mm, length/breadth ratio of 2.5 to 3.0 mm; Short (SS) grain segment with grain type of short slender, length of less than 6 mm, length/breadth ratio of larger than 3. All parental lines were derived from the indica rice pool with some introgressions from the japonica group. The lines were grouped into restorer (male) and maintainer (female) lines, because hybrids are produced based on a cytoplasmic male sterility (CMS) system. Most lines were used specifically for a certain market segment and only 17 lines were used as parents across different market segments (Additional file 1: Figure S5). The total number of unique lines amounted to 270, from which 262 were used for genotyping.

For the hybrid production in segment LS, 109 males and 13 females were used, segment MM consisted of 95 male and 16 female parents and in segment SS crosses were made between 47 male and 10 female lines (Additional file 1: Figure S4). The lower number of female than male lines is because it is easier to breed restorer than maintainer lines. The parental lines were crossed using an unbalanced factorial mating design. Phenotypic data comprised of 625, 935, and 400 hybrids for the LS, MM, and SS segment, respectively.

### Field trials

The phenotypic data is based on grain yield trials conducted in the year 2014. The hybrids were evaluated in three, two, and four locations for LS, MM, and SS, respectively. The locations were Faizabad (FZB, 26.78°N; 82.13°E; loam, 97 m a.s.l.), Hyderabad (HYD, 17.44°N; 78.11°E, black loam, 569 m a.s.l.), Dhantori (DHN, 28.2°N; 75.45°E, loam, 257 m a.s.l.), Mysore (MYS, 12.5°N; 76.67°E, loam, 709 m a.s.l.), and Raipur (RPR, 21.19°N; 81.56°E, loam, 298 m a.s.l.). In all locations, genotypes were sown in nursery beds and 30 days aged seedlings were manually transplanted in the puddled main field conditions.

The experimental designs followed augmented designs including trials and blocks within replicated checks, but unreplicated entries. Restrictions in hybrid seed production and restricted budget were the main reason for choosing an augmented design with replicated checks, but unreplicated hybrids. The design of a field experiment is graphically illustrated for location HYD and segment MM (Additional file 1: Figure S6). The field experiments in the different locations were structured into trials. Every trial was further split into blocks including entries and five to seven checks. Plot size ranged from 3.6 m^2^ to 4.2 m^2^ and transplanting density was 33 seedlings m^−2^ using one seedling per hill.

### Genomic marker data

In total 262 parental lines were genotyped with a 6 K SNP array based on an Illumina Infinium assay (Yu et al. 2014). After excluding markers with minor allele frequency below 5%, 5,221 high-quality SNP markers were left for further analyses. For 612, 701, and 242 hybrids genotypic information was available for the LS, MM, and SS segment, respectively.

### Statistical analyses of field trials

We analyzed every market segment independently. The variance components were estimated fitting the following model:

$$ {y}_{j k mn}=\mu +{m}_j+{g}_j+{l}_k+{t}_{k m}+{b}_{k m n}+{(gl)}_{j k}+{\varepsilon}_{j k mn}, $$

where *y*_*jkmn*_ is the grain yield of *j* ^th^ genotype in *n* ^th^ block, *m* ^th^ trial and *k* ^th^ location, *μ* is the overall mean, *m*_*i*_ is an effect for maturity of the observed genotype; *g*_*j*_ refers to the effect of the *j* ^th^ genotype, *l*_*k*_ to the effects of the ${k}^{{}^{\mathrm{th}}}$ location, *t*_*km*_ to the effect of the *m* ^th^ trial at the ${k}^{{}^{\mathrm{th}}}$ location, *b*_*kmn*_ to the effect of the ${n}^{{}^{\mathrm{th}}}$ block at the *m* ^th^ trial at the ${k}^{{}^{\mathrm{th}}}$ location, (*gl*)_*jk*_ to the interaction effect between the ${j}^{{}^{\mathrm{th}}}$ genotype and the ${k}^{{}^{\mathrm{th}}}$ location and *ε*_*jkmn*_ to the residual. All effects except *m*_*i*_ were modeled as random. The estimated variance components were used to calculate the broad-sense heritability as:

$$ {h}^2=\frac{\sigma_{Genotype}^2}{\sigma_{Phenotype}^2}=\frac{\sigma_{Genotype}^2}{\sigma_{Genotype}^2+\frac{\sigma_{GxL}^2}{No.\  of\  location}+\frac{\sigma_{error}^2}{No.\  of\  location \times No.\  of\  replicates}} $$

The genotypic variance was further decomposed into the variance due to general (GCA) and specific combining ability effects (SCA). Significance of variance components were tested using a likelihood-ratio test. We used the above outlined model and assumed fixed genotype effects to obtain the best linear unbiased estimations (BLUEs) of every genotype (Additional file 2). All described statistical approaches were conducted using R (The R Core Team 2016) and linear mixed models were implemented with the package ASReml-R (Gilmour et al. 2009).

### Analyses of population structure

The genomic data were used to estimate the allele frequency of every marker. Linkage disequilibrium (LD) was assessed by the LD measure r^2^ (Weir 1996). Rogers’ distances (Rogers 1972) among all pairs of parental lines were calculated. The matrix of Rogers’ distances was used to perform principal coordinate analyses (Gower 1966).

### Prediction of hybrid performance

The genome-wide prediction models make use of all hybrids for which genotypic and phenotypic information were available, i.e., 612, 701, and 242 hybrids for the LS, MM, and SS segment, respectively. For genome-wide prediction, we implemented ridge regression best linear unbiased prediction (RR-BLUP), modeling both additive and dominance effects as:

$$ Y={1}_n\mu +{Z}_A a+{Z}_D d+ e. $$

*Y* refers to BLUEs of the hybrids for grain yield. Vector 1_*n*_ includes only ones and its element number is equal to the number of *n* hybrids; *μ* refers to the overall mean. Design matrices *Z*_*A*_ and *Z*_*D*_ have a dimension of *n* × *m*, where *m* is the number of markers and were defined using the F_∞_ metric (Zhao et al. 2013). The vector *e* includes residual values for the single cross combinations. Estimations or predictions of *a*, *d*, and *μ* were done by implementing Henderson’s mixed model equation (Henderson 1984). Normal distribution and constant variance for the additive and dominance effects were assumed (Zhao et al. 2013).

The accuracy of the prediction of grain yield was evaluated using cross-validations, which entails the division of the total population into estimation and test populations. Marker effects were estimated in the estimation population and used to predict the performance of hybrids in the test population. Since relatedness strongly influences prediction accuracy (Habier et al. 2007), a cross-validation strategy was used considering three test populations with varying degree of relatedness to the estimation population (Additional file 1: Figure S3): Test set T2 most closely related to the estimation set included only hybrids derived from the same parents as the hybrids that had been evaluated, while the less related test set T1 included hybrids sharing one parent with the hybrids in the estimation set and the least related test set T0 included only hybrids having no parents in common with the estimation set. Prediction ability was estimated as Pearson’s correlation coefficient between the observed and the predicted hybrid performance.

### Identification of heterotic groups

The search of heterotic patterns maximizing the hybrid performance was performed based on the predicted hybrid performances using a simulated annealing algorithm. The implementation of the simulated annealing algorithm is described in detail by Zhao et al. (2015). We assumed the same group size for two matching heterotic groups and performed the search for increasing group sizes from two to 20.

### Assessing the short- and long-term success of heterotic groups

Beside the hybrid performance as a short-term success parameter, theoretical selection limit (Zhao et al. 2015) and genetic representativeness describing long-term success were determined to evaluate the performance of the identified heterotic groups. The genetic representativeness gives the proportion of specific genomes within the full population represented by the identified heterotic groups (Druet et al. 2014). Representativeness of two specific heterotic groups depends from the additive relationship within heterotic groups beside additive relationship between the full population and heterotic groups and the genome proportion of heterotic groups. Additive relationship was calculated as one minus the Rogers’ distance values of parental pairs (Melchinger et al. 1991). The final representativeness for two matching heterotic groups was the result of the mean value of all parental lines included in the selected heterotic patterns (Zhao et al. 2015). Theoretical selection limit is closely linked to the concept of maximal long-term selection response (Walsh and Lynch 2014) and represents the maximum hybrid performance, which can be reached by reciprocal recurrent selection based on the identified heterotic groups performing an infinite number of selection cycles. We assumed absence of migration, mutation, and epistasis for estimating of theoretical selection limit based on the predicted marker effects (Zhao et al. 2015).

## Additional files

Additional file 1: for Genome-based identification of heterotic. **Figure S1.** Decay of linkage disequilibrium measured using r^2^ with physical map distance. **Figure S2.** Principal coordinate analysis of all parental lines for market segments LS, MM, and SS. **Figure S3.** Applied cross validation scenario exemplifying the selected fractions for market segment MM. **Figure S4.** Unbalanced factorial crossing designs for market segment (A) LS, (B) MM, and (C) SS. **Figure S5.** Venn diagram of overlapping parental lines of the hybrid evaluation trials in market segments LS, MM, and SS. **Figure S6.** Experimental design and distribution of checks for hybrid experiment of market segment MM at location HYD. Orange and black lines represent the size of trials and blocks, respectively. White plots were not phenotyped. **Table S1.** Composition of heterotic groups selected with the simulated annealing algorithm as well as the average hybrid performance between both heterotic groups (Inter) for the market segments LS, MM, and SS. **Table S2.** Composition of estimation and test populations for market segment LS, MM, and SS. (DOCX 1811 kb)

Additional file 2: Best linear unbiased estimations for hybrid grain yield experiments in market segments LS, MM, and SS. (XLSX 76 kb)
