# Supplementary material for: Genome-Based Identification of Heterotic Patterns in Rice
Source: Rice (N Y). 2017 May 19;10:22. doi: 10.1186/s12284-017-0163-4 (PMC5438337; doi:10.1186/s12284-017-0163-4)
Supplement: Supplementary file 1 — for Genome-based identification of heterotic. Figure S1. Decay of linkage disequilibrium measured using r2 with physical map distance. Figure S2. Principal coordinate analysis of all parental lines for market segments LS, MM, and SS. Figure S3. Applied cross validation scenario exemplifying the selected fractions for market segment MM. Figure S4. Unbalanced factorial crossing designs for market segment (A) LS, (B) MM, and (C) SS. Figure S5. Venn diagram of overlapping parental lines of the hybrid evaluation trials in market segments LS, MM, and SS. Figure S6. Experimental design and distribution of checks for hybrid experiment of market segment MM at location HYD. Orange and black lines represent the size of trials and blocks, respectively. White plots were not phenotyped. Table S1. Composition of heterotic groups selected with the simulated annealing algorithm as well as the average hybrid performance between both heterotic groups (Inter) for the market segments LS, MM, and SS. Table S2. Composition of estimation and test populations for market segment LS, MM, and SS. (DOCX 1811 kb) [file 12284_2017_163_MOESM1_ESM.docx]

**Additional file 1 for Genome-based identification of heterotic patterns in rice**

Ulrike Beukert^1^, Zuo Li^1^, Guozheng Liu^1^, Yusheng Zhao^1^, Nadhigade Ramachandra^2^, Vilson Mirdita^3^, Fabiano Pita^4^, Klaus Pillen^5^, Jochen Christoph Reif^1*^

^1^Department of Breeding Research, Leibniz Institute of Plant Genetics and Crop Plant Research (IPK) Gatersleben, Corrensstraße 3, 06466 Stadt Seeland, Germany

^2^ Bayer Bioscience, 500081 Hyderabad, India

^3^ European Wheat Breeding Center, Bayer Crop Science, Am Schwabenplan 8, 06466 Stadt Seeland, Germany

^4^ Biometrics and Breeding Research US, Bayer Crop Science, 407 Davis Dr., Morrisville, North Carolina, USA

^5^ K. Pillen, Chair of Plant Breeding, Martin-Luther-University Halle-Wittenberg, Betty-Heimann-Str. 3, 06120 Halle/Saale, Germany.

* Correspondence: [reif@ipk-gatersleben.de](mailto:reif@ipk-gatersleben.de)

**
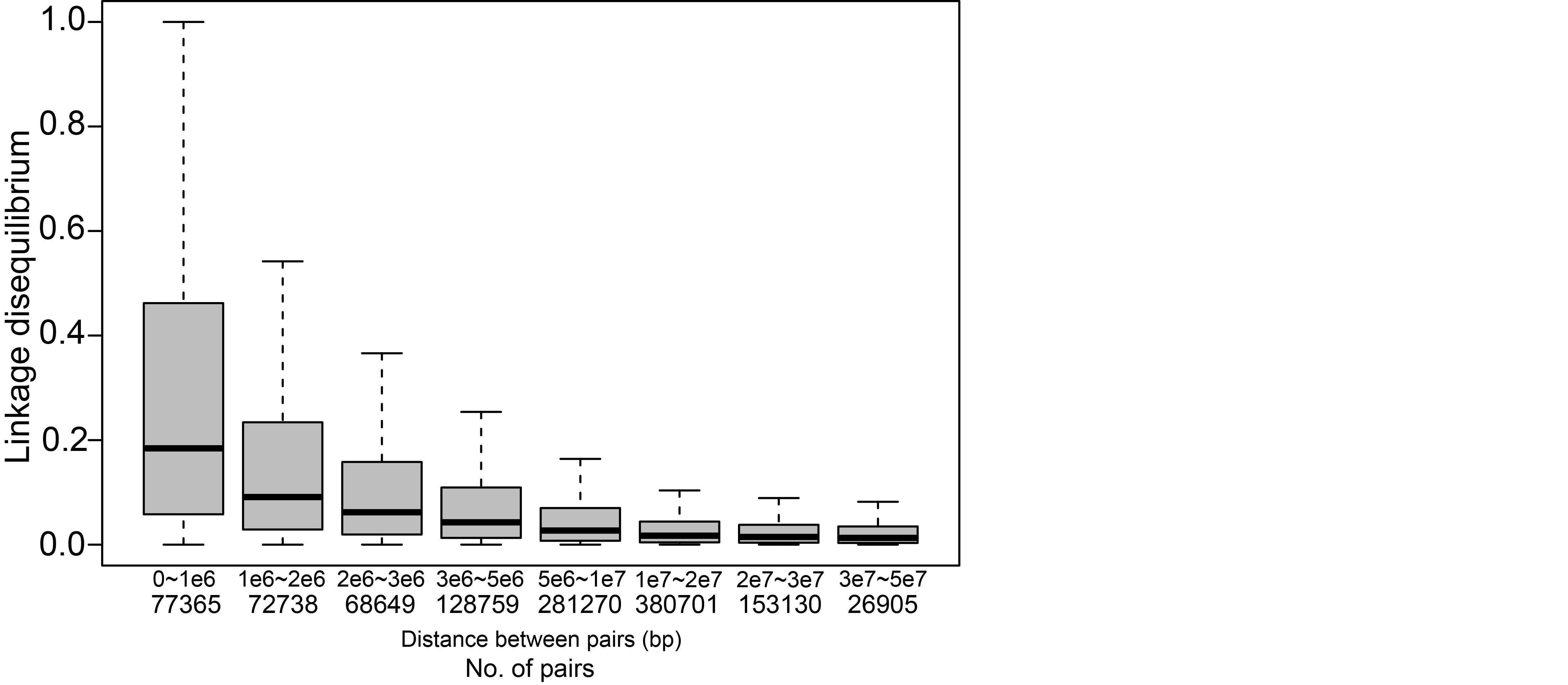
Fig. S1** Decay of linkage disequilibrium measured using r² with physical map distance.


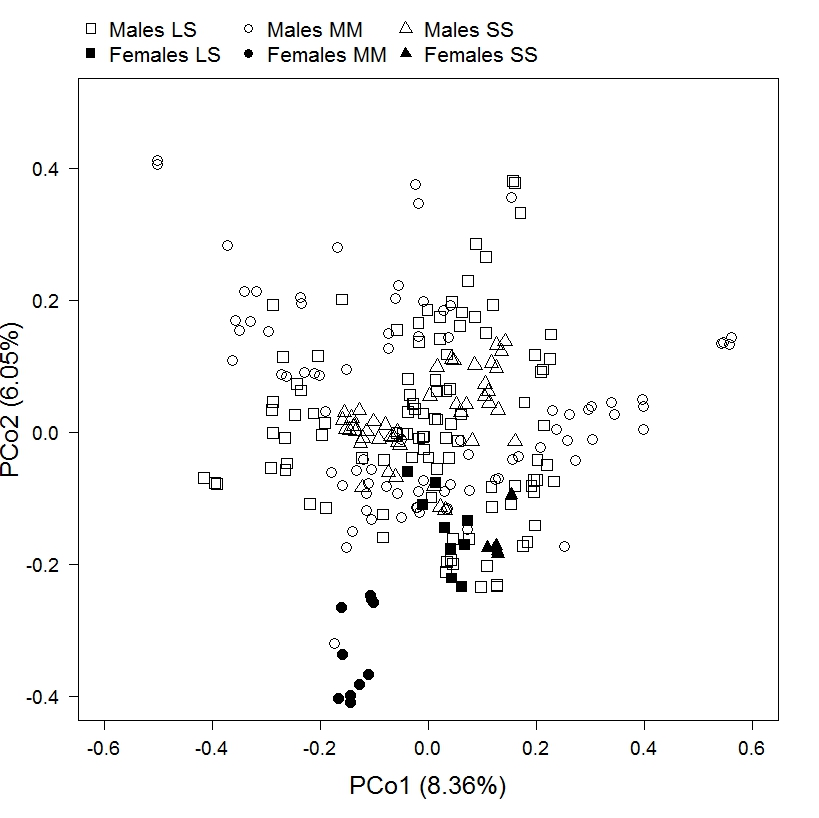


**Fig. S2** Principal coordinate analysis of all parental lines for market segments LS, MM, and SS.


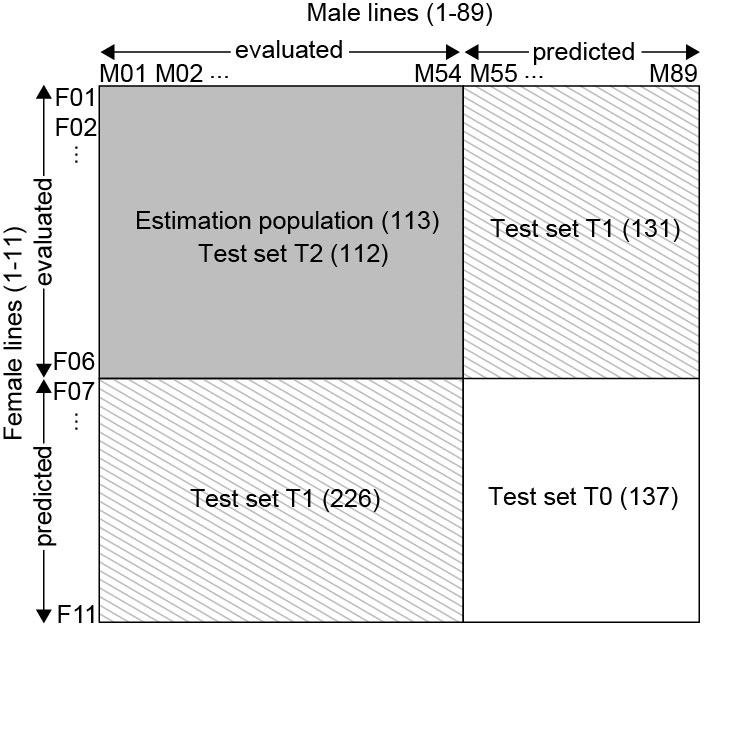


**Fig. S****3** Applied cross validation scenario exemplifying the selected fractions for market segment MM.

**
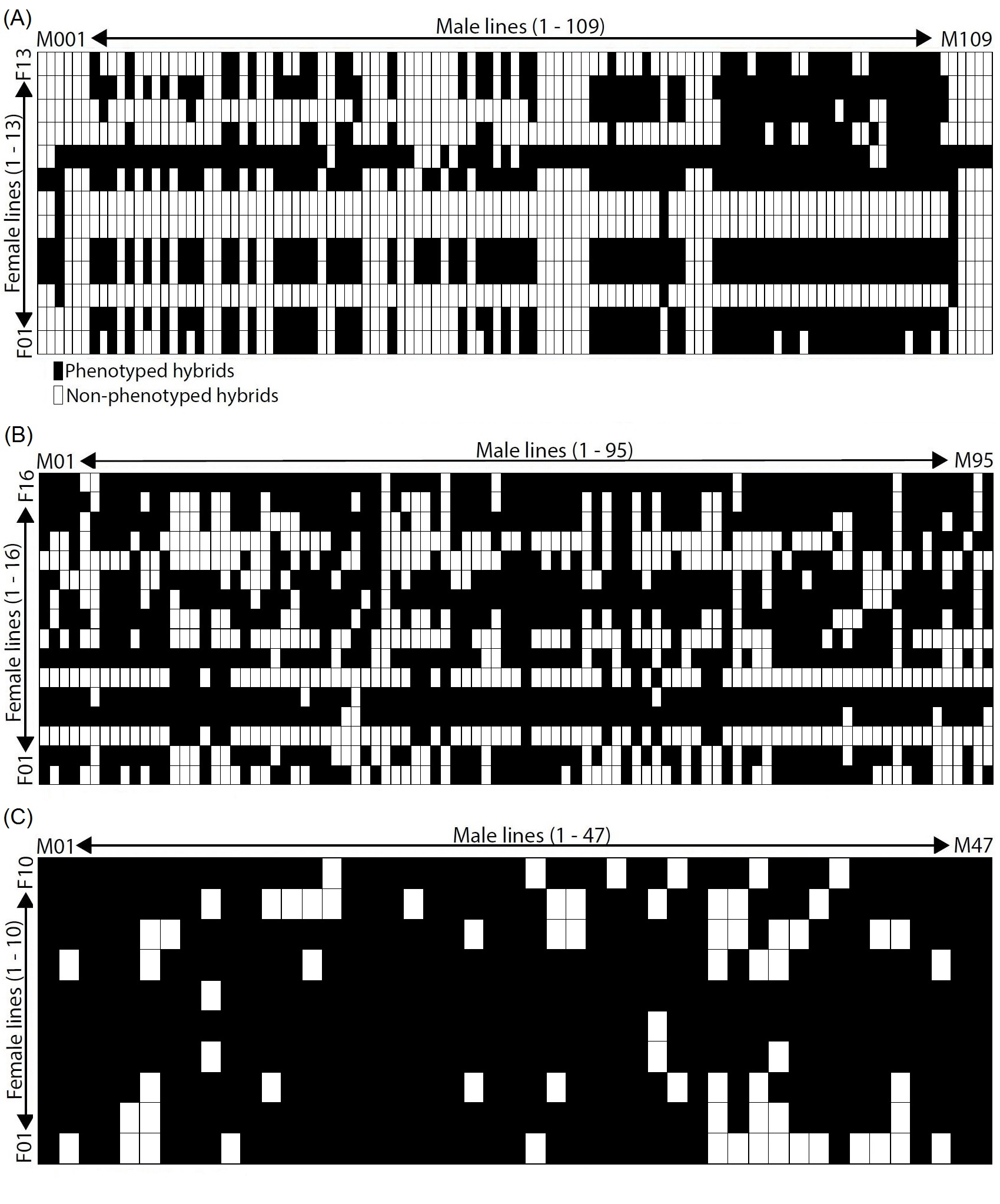
**

**Fig. S4** Unbalanced factorial crossing designs for market segment (A) LS, (B) MM, and (C) SS.


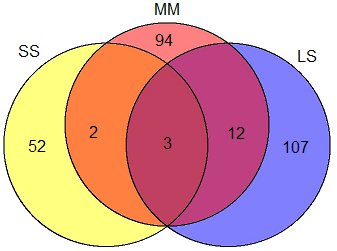


Fig. S5 Venn diagram of overlapping parental lines of the hybrid evaluation trials in market segments LS, MM, and SS.


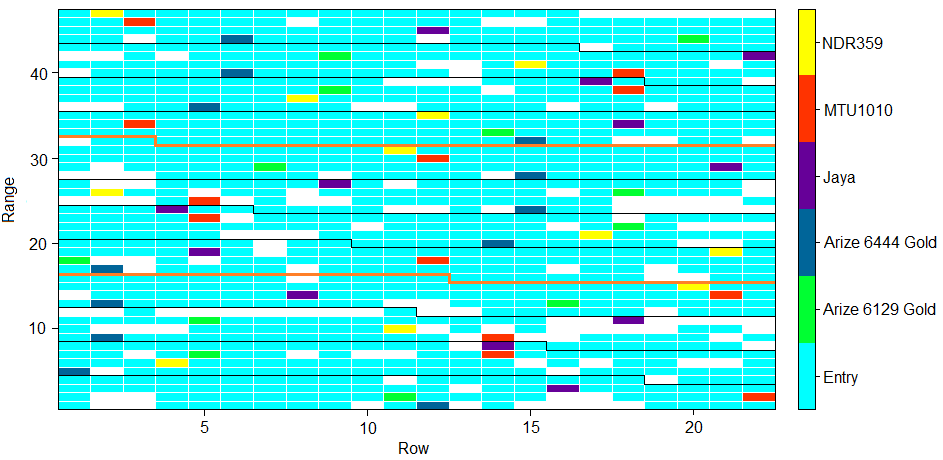


Fig. S6 Experimental design and distribution of checks for hybrid experiment of market segment MM at location HYD. Orange and black lines represent the size of trials and blocks, respectively. White plots were not phenotyped.

Table S1 Composition of heterotic groups selected with the simulated annealing algorithm as well as the average hybrid performance between both heterotic groups (Inter) for the market segments LS, MM, and SS.

| Segment | Group  size | Selected lines  Group 1 | Selected lines  Group 2 | Inter  (Mg ha^-1^) |
| --- | --- | --- | --- | --- |
| LS | 2 | IPL1104 IPL1103 | IPL1109 IPL1082 | 12.21 |
| LS | 4 | IPL1104 IPL1105IPL1103 IPL1100 | C1130 IPL1109C1129 IPL1082 | 11.82 |
| LS | 6 | IPL1100 IPL1104 IPL1105 IPL1103 IPL1026 IPL1006 | IPL1082 IPL1109 IPL1065 C1129 IPL1056 C1130 | 11.63 |
| LS | 8 | IPL1056 IPL1100 IPL1026 IPL1105 IPL1104 IPL1106 IPL1103 IPL1006 | IPL1034 IPL1054 IPL1109 C1129 IPL1065 C1130 IPL1059 IPL1082 | 11.49 |
| LS | 10 | IPL1106 IPL1109 IPL1022 IPL1104 IPL1105 IPL1103 IPL1100 IPL1026 IPL1056 IPL1006 | IPL1034 C1112 IPL1082 C1126 C1129 IPL1054 IPL1065 IPL1048 IPL1059 C1130 | 11.35 |
| LS | 12 | IPL1100 IPL1105 IPL1109 IPL1104 IPL1056 IPL1006 IPL1043 IPL1022 IPL1023 IPL1106 IPL1103 IPL1026 | IPL1065 IPL1054 IPL1048 C1140 IPL1059 C1126 IPL1034 C1130 IPL1040 C1129 C1112 IPL1082 | 11.24 |
| LS | 14 | IPL1109 IPL1103 IPL1026 IPL1043 IPL1056 IPL1105 IPL1006 IPL1023 IPL1104 IPL1099 IPL1002 IPL1022 IPL1106 IPL1100 | IPL1048 IPL1065 IPL1027 IPL1067 IPL1082 IPL1040 IPL1059 C1130 C1129 IPL1034 C1112 C1126 IPL1054 C1140 | 11.14 |
| LS | 16 | IPL1026 IPL1104 IPL1006 IPL1105 IPL1100 IPL1043 IPL1106 IPL1056 IPL1089 IPL1099 IPL1107 IPL1014 IPL1098 IPL1002 IPL1022 IPL1103 | IPL1048 C1140 IPL1034 IPL1023 IPL1059 C1112 IPL1082 IPL1067 IPL1065 C1130 IPL1040 IPL1027 C1126 IPL1109 IPL1054 C1129 | 11.05 |
| LS | 18 | IPL1006 IPL1022 IPL1056 IPL1035 IPL1099 IPL1043 IPL1026 IPL1085 IPL1103 IPL1002 IPL1100 IPL1098 IPL1104 IPL1106 IPL1014 IPL1105 IPL1089 IPL1107 | C1140 IPL1048 IPL1082 IPL1109 IPL1027 IPL1059 C1129 IPL1041 IPL1040 IPL1023 C1126 IPL1065 IPL1034 C1130 IPL1054 IPL1067 C1112 IPL1010 | 10.97 |
| LS | 20 | IPM1016 IPL1038 IPL1107 IPL1090 IPL1043 IPL1014 IPL1022 IPL1106 IPL1100 IPL1056 IPL1099 IPL1103 IPL1105 IPL1104 IPL1089 IPL1026 IPL1085 IPL1006 | C1112IPL1034 IPL1095 IPL1082 IPL1035 IPL1041 IPL1027 IPL1040 IPL1059 IPL1067 IPL1023 IPL1065 IPL1048 C1130 IPL1010 IPL1054 C1126 IPL1109 | 10.90 |
| LS | 20 | IPL1098 IPL1002 | C1129 C1140 | 10.90 |
| MM | 2 | IPM1039 IPM1057 | IPM1076 IPM1041 | 11.36 |
| MM | 4 | IPM1077 IPM1057IPM1015 IPM1039 | IPM1007 IPM1076 IPM1041 IPM1069 | 10.95 |
| MM | 6 | IPM1077 IPM1015 IPM1049 IPM1045 IPM1039 IPM1057 | IPM1056 IPM1007 IPM1041 IPM1060 IPM1069 IPM1076 | 10.75 |
| MM | 8 | IPM1045 IPM1077 IPM1057 IPM1065 IPM1015 IPM1049 IPM1039 IPM1013 | IPM1003 IPM1001 IPM1007IPM1076 IPM1056 IPM1060 IPM1041 IPM1069 | 10.62 |
| MM | 10 | IPM1039 IPM1077 IPM1065 IPM1015 IPM1045 IPM1013 IPM1066 IPM1047 IPM1049 IPM1057 | IPM1076 IPM1056 IPM1041 IPM1060 IPM1069 IPM1001 IPM1007 IPM1003 IPM1023 IPM1074 | 10.53 |
| MM | 12 | IPM1049 IPM1066 IPM1057 IPM1015 IPM1038 IPM1039 IPM1065 IPM1047 IPM1077 IPM1013 IPM1045 IPM1060 | IPM1007 IPM1069 IPM1053 IPM1001 IPM1074 IPM1054 IPM1076 IPM1041 IPM1003 IPM1023 IPM1031 IPM1056 | 10.45 |
| MM | 14 | IPM1077 IPM1049 IPM1013 IPM1015 IPM1045 IPM1038 IPM1057 IPM1039 IPM1065 IPM1002 IPM1060 IPM1071 IPM1047 IPM1066 | IPM1018 IPM1056 IPM1031 IPM1069 IPM1003 IPM1053 IPM1076 IPM1074 IPM1007 IPM1004 IPM1054 IPM1041 IPM1001 IPM1023 | 10.38 |
| MM | 16 | IPM1063 IPM1013 IPM1045 IPM1039 IPM1031 IPM1065 IPM1077 IPM1060 IPM1038 IPM1071 IPM1049 IPM1066 IPM1002 IPM1057 IPM1015 IPM1047 | IPM1012 IPM1001 IPM1074 IPM1018 IPM1069 IPM1041 IPM1056 IPM1054 IPM1023 IPM1009 IPM1076 IPM1004 IPM1003 IPM1007 IPM1017 IPM1053 | 10.31 |
| MM | 18 | IPM1045 IPM1048 IPM1057 IPM1038 IPM1013 IPM1060 IPM1031 IPM1066 IPM1051 IPM1015 IPM1049 IPM1039 IPM1077 IPM1071 IPM1063 IPM1047 IPM1065 IPM1002 | IPM1004 IPM1018 IPM1054 IPM1074 IPM1059 IPM1056 IPM1076 IPM1041 IPM1053 IPM1007 IPM1003 IPM1023 IPM1069 IPM1009 IPM1012 IPM1017 IPM1055 IPM1001 | 10.26 |
| MM | 20 | IPM1077 IPM1060 IPM1049 IPM1047 IPM1051 IPM1015 IPM1063 IPM1038 IPM1002 IPM1066 IPM1057 IPM1045 IPM1039 IPM1048 IPM1070 IPM1041 IPM1035 IPM1065 IPM1013 IPM1071 | IPM1008 IPM1003 IPM1031 IPM1076 IPM1069 IPM1004 IPM1007 IPM1054 IPM1050 IPM1001 IPM1017 IPM1009 IPM1059 IPM1018 IPM1012 IPM1053 IPM1056 IPM1055 IPM1023 IPM1074 | 10.19 |
| SS | 2 | IPS1014 IPS1026 | C1109 IPS1012 | 8.25 |
| SS | 4 | IPS1014 IPS1002 IPS1032 IPS1026 | IPS1012 C1109 IPS1013 IPS1006 | 8.16 |
| SS | 6 | IPS1002 IPS1014 IPS1026 IPS1032 IPS1031 IPS1028 | IPS1007 IPS1013 C1111 C1109 IPS1006 IPS1012 | 8.09 |
| SS | 8 | IPS1002IPS1026 IPS1032 IPS1010 IPS1006 IPS1014 | IPS1012 C1111 C1099 IPS1013 IPS1009 IPS1011 | 8.00 |
| SS | 8 | IPS1028 IPS1031 | IPS1007 C1109 | 8.00 |
| SS | 10 | IPS1002 IPS1031 IPS1032 IPS1006 IPS1010 IPS1028 IPS1029 IPS1014 IPS1042 IPS1026 | IPS1013 IPS1011 IPS1025 C1111 IPS1007 C1099 IPS1005 C1109 IPS1009 IPS1012 | 7.92 |
| SS | 12 | IPS1042 IPS1010 IPS1026 IPS1028 IPS1029 IPS1027 IPS1005 IPS1002 IPS1032 IPS1006 IPS1031 IPS1014 | IPS1007 IPS1011 IPS1012 IPS1009 IPS1025 C1099 IPS1013 C1102 C1109 IPS1033 C1111 IPS1037 | 7.83 |
| SS | 14 | IPS1031 IPS1011 IPS1028 IPS1027 IPS1029 IPS1002 IPS1042 IPS1032 IPS1009 IPS1026 IPS1014 IPS1006 IPS1025 IPS1010 | IPS1013 IPS1036 IPS1012 IPS1033 IPS1007 IPS1005 IPS1035 IPS1037 C1109 C1111 IPS1047 IPS1020 C1102 C1099 | 7.74 |
| SS | 16 | IPS1003 IPS1032 IPS1011 IPS1031 IPS1029 IPS1027 IPS1009 IPS1025 IPS1010 IPS1001 IPS1026 IPS1042 IPS1002 IPS1007 IPS1014 IPS1028 | IPS1015 IPS1005 IPS1036 IPS1037 IPS1006 IPS1047 C1109IPS1035 IPS1008 IPS1033 C1111 IPS1012 C1099 IPS1013 C1102 IPS1020 | 7.67 |
| SS | 18 | IPS1010 IPS1002 IPS1026 IPS1042 IPS1029 IPS1014 IPS104, IPS1011 IPS1031 IPS1006 IPS1027 IPS1025 IPS1028 IPS1033 IPS1007 IPS1001 IPS1009 IPS1032 | C1099 IPS1015 C1111 IPS1005 IPS1017 IPS1020 IPS1016 IPS1047 C1109 IPS1003 IPS1044 IPS1037 C1102 IPS1008 IPS1013 IPS1036 IPS1035 IPS1012 | 7.61 |
| SS | 20 | IPS1014 IPS1031 IPS1025 IPS1035 IPS1019 IPS1016 IPS1010 IPS1001 IPS1008 IPS1041 IPS1006 IPS1032 IPS1009 IPS1002 IPS1027 IPS1028 IPS1042 IPS1011 IPS1029 IPS1026 | C1111 IPS1020 IPS1036 C1099 IPS1039 IPS1013 C1109 IPS1012 IPS1015 IPS1005 IPS1037 IPS1044 IPS1003 C1102 IPS1047 IPS1017 IPS1033 IPS1007 IPS1018 IPS1045 | 7.54 |

**Table S2** Composition of estimation and test populations for market segment LS, MM, and SS.

|  | LS | MM | SS |
| --- | --- | --- | --- |
| *Estimation population* |  |  |  |
| No. of hybrids | 193 | 113 | 115 |
| No. of female parents | 7 | 6 | 5 |
| No. of male parents | 70 | 54 | 32 |
| *Test population T0* |  |  |  |
| No. of hybrids | 62 | 137 | 11 |
| No. of female parents | 3 | 5 | 1 |
| No. of male parents | 24 | 35 | 11 |
| *Test population T1* |  |  |  |
| No. of hybrids | 265 | 339 | 88 |
| No. of female parents | 10 | 11 | 6 |
| No. of male parents | 75 | 88 | 39 |
| *Test population T2* |  |  |  |
| No. of hybrids | 92 | 112 | 28 |
| No. of female parents | 7 | 6 | 5 |
| No. of male parents | 41 | 49 | 20 |
